# Mechanical and microstructural characteristics of calcareous sand reinforced by MICP-hollow fiber membrane treatment

**DOI:** 10.3389/fmicb.2026.1846992

**Published:** 2026-05-20

**Authors:** Yongchang Yang, Shuai Zhang, Jun Hu, Dongling Zeng, Xiang Xue, Yufei Yang

**Affiliations:** 1 Hainan Key Laboratory of Marine Geological Resources and Environment, Haikou, China; 2 Hainan Institute of Hydrological and Geological Engineering Exploration, Haikou, China; 3 School of Information and Communication Engineering, Hainan University, Haikou, China; 4 Department of Infrastructure Engineering, The University of Melbourne, Parkville, VIC, Australia; 5 School of Civil Engineering and Architecture, Hainan University, Haikou, China; 6 State Key Laboratory of Tropic Ocean Engineering Materials and Materials Evaluation, Hainan University, Haikou, China; 7 Collaborative Innovation Center of Marine Science and Technology, Hainan University, Haikou, China; 8 Sanya Hydrogeological Engineering Geological Survey Institute, Sanya, China; 9 Sanya Construction Engineering Quality Testing Center, Sanya, China

**Keywords:** bacterial retention rate, calcareous sand, hollow fiber membrane, mechanical properties, MICP, microscopic mechanisms

## Abstract

**Introduction:**

To address the engineering challenges of low strength and high crushability of calcareous sand in South China Sea islands and reefs, this study innovatively combines microbially induced calcium carbonate precipitation (MICP) technology with hollow fiber membranes.

**Methods:**

The effects of different hollow fiber membrane contents (0.2%–1%) and lengths (5–20 mm) on calcareous sand reinforcement were systematically investigated through physical property tests, mechanical property tests, and SEM analysis.

**Results:**

Results demonstrate that hollow fiber membranes (HFM) significantly enhance MICP effectiveness. Under optimal conditions (0.6% content, 10 mm length), calcium carbonate generation rate, bacterial retention rate, unconfined compressive strength, and cohesion improved by 37.7, 129.3, 48.13, and 64.49%, respectively. Microscopic analysis reveals triple reinforcement mechanisms: MICP strengthening through bridging, cementation, and coating effects; spatial constraint from hollow fiber membrane networks; and enhanced interfacial bonding from surface crystallization. Significant interactions exist between membrane content and length, longer membranes excel at low contents while shorter membranes avoid agglomeration at high contents.

**Discussion:**

Under laboratory conditions, the HFM–MICP combination improves the strength and microstructural performance of calcareous sand, indicating potential relevance to the reinforcement of calcareous soils in marine geotechnical settings.

## 1 Introduction

Calcareous sand is a major component of coastal zones and island reefs. It has high porosity and low bearing capacity. The particles are easily crushed under pressure. This leads to coastal zone degradation under long-term hydrodynamic erosion from seawater. It also poses safety risks to marine engineering structures built on such foundations (Kou et al., 2023). With the rapid development of the marine economy, there is an urgent need for foundation reinforcement in coastal zones and island reef areas. This will improve the physical and mechanical properties of calcareous sand regions. Traditional reinforcement methods include chemical stabilization, geotextiles, and cement stabilization. These methods have been widely applied and play important roles in improving the physical and mechanical properties of calcareous sand. However, they have environmental drawbacks such as high energy consumption, high carbon emissions, and chemical pollution (Li et al., 2022).

Microbially induced calcium carbonate precipitation (MICP) technology is a novel ground treatment technique. It utilizes specific microorganisms widely present in nature. These microorganisms produce calcium carbonate precipitation through their metabolism to cement soil particles. MICP has advantages of environmental friendliness and ultra-low energy consumption. It shows broad application prospects in geotechnical engineering. Existing studies demonstrate that MICP technology can be effectively applied to sand reinforcement (Zhang et al., 2022; Lin et al., 2020), rock or concrete crack repair (Cui et al., 2025; Intarasoontron et al., 2021; Zhang et al., 2024; Zhou et al., 2024), contaminated soil remediation (Kumari et al., 2014; Kang et al., 2014), and slope erosion resistance (Gowthaman et al., 2022; Jiang et al., 2023; Moqsud and Gochi, 2023).

Many factors influence MICP reinforcement effectiveness. Regarding temperature, Wang et al. (2023) found that calcium carbonate crystals formed at different temperatures vary in size and type. Low temperatures favor vaterite formation while high temperatures favor calcite formation. Concerning bacterial solution properties, Cheng et al. (2016) discovered that lower urease activity creates more effective crystalline precipitation patterns under the same calcium carbonate precipitation amount. For cementing solution concentration, Qabany and Soga (2013) found that lower concentrations such as 0.1 M and 0.25 M achieve more uniform calcium carbonate precipitation. Regarding injection methods, Cheng et al. (2019) found that reducing bacterial solution pH can prevent clogging from bioflocculation and achieve relatively uniform MICP reinforcement. These studies provide important references for optimizing MICP technology. However, although MICP technology can effectively improve soil strength, it has limitations of poor soil toughness and susceptibility to brittle failure (Xie et al., 2019; Cui et al., 2017).

Fiber reinforcement technology provides an effective solution to improve the mechanical properties of MICP-treated soil. Studies show that incorporating fibers into soil can effectively improve mechanical properties such as wet-dry cycle durability, shear strength, and stiffness (Consoli et al., 2019; Kanchi et al., 2015). Researchers have combined traditional fiber materials with MICP technology. Lv et al. (2021) investigated the reinforcement effects of combining polypropylene fibers, basalt fibers, and carbon fibers with MICP. They found that fiber incorporation provides favorable space for bacterial colonization. This increases calcium carbonate precipitation and transforms the failure mode of cured sand from brittle to ductile failure. Choi et al. (2016) found that polyvinyl alcohol fibers can significantly reduce the brittleness of MICP-cured specimens. The strength ratio decreased by 50%. Wang et al. (2022) studied MICP combined with polypropylene fiber modification of calcareous sand. Their results showed that fiber addition provides broader space for microbial attachment and promotes calcium carbonate crystal formation. Dynamic strain and dynamic pore pressure decreased by 74.32 and 74.18%, respectively. Liu et al. (2019) found that adding appropriate amounts of fiber can significantly improve the ductility of MICP-treated sand. This helps resist harsh environmental conditions such as wet-dry cycles and freeze–thaw cycles (Li et al., 2021).

Hollow fiber membranes represent an advanced membrane technology product. They are the result of combining functional fiber materials with separation membrane technology. This technology shows significant advantages including high-density packing per unit volume, stable physicochemical properties, excellent filtration efficiency, and relatively low manufacturing costs (Xiao et al., 2021). The hollow fiber walls are covered with micropores. Suspended solids, bacteria, and other macromolecular organic compounds larger than the ultrafiltration membrane pore size are retained. This achieves purification effects (Khelifi et al., 2025). Hollow fiber membranes were first applied in wastewater treatment (Mohmed et al., 2025). Today, their applications have expanded to petrochemicals (Mohdee et al., 2022), hemodialysis (Yang et al., 2024), drug purification (Wan and Chen, 2025), and other fields. They are a key development direction for strategic emerging industries. Combining hollow fiber membranes with MICP technology offers unique benefits. Their microporous structure can provide more attachment sites for bacteria, and they can improve bacterial distribution uniformity in soil through their excellent retention capacity. Recent advances in MICP technology have explored various optimization strategies, including the chemical regulation of precipitation kinetics (e.g., Hu et al., 2025) and the incorporation of solid fibers for matrix reinforcement (e.g., Chang et al., 2026). However, the specific application of permeable hollow fiber membranes (HFMs) for the structural reinforcement of particulate soils, such as calcareous sand, remains largely unexplored. Unlike conventional solid fibers that act primarily as macroscopic bridging inclusions, the ~0.1 μm wall pores of HFMs create a pronounced size-exclusion effect against *Sporosarcina pasteurii* cells (S.pasteurii, formerly known as *Bacillus pasteurii*; 2–3 μm length). This unique microstructural feature enables the membrane to concurrently serve as a physical bacterial retainer, a multi-scale spatial constraint network, and a heterogeneous nucleation substrate for CaCO₃.

Based on the above background, this study innovatively combines MICP technology with hollow fiber membranes. The objective is to reinforce calcareous sand in South China Sea islands and reefs. This study investigates the effects of different contents (0.2–1%) and lengths (5–20 mm) of hollow fiber membranes on microbial treatment of calcareous sand. The aim is to determine the optimal fiber membrane ratio and treatment method. This will provide technical support and reference for MICP technology applications in South China Sea island and reef calcareous sand reinforcement projects. This research not only fills the research gap in combining MICP with hollow fiber membranes but also reveals the reinforcement mechanisms from physical-mechanical-microscopic multi-scale perspectives. It has important significance for promoting the engineering applications of bio-geotechnical technology.

## 2 Materials and methods

### 2.1 Test materials

The test sand was collected from an island in Sansha City, Hainan Province. The original sand samples were washed, dried, and cooled to remove impurities. The mineral composition of the sand was determined through X-ray diffraction (XRD) and X-ray fluorescence spectroscopy (XRF) comprehensive tests. Test results show that the calcareous sand composition is presented in Table 1. Basic physical property indices determined according to the “Standard for Soil Test Methods” (GB/T 50123–2019) are shown in Table 2. The particle size distribution curve indicates that this sand belongs to poorly graded soil. Microstructural observation shows that pores exist on the sand particle surfaces. These characteristics provide favorable conditions for microbial attachment and calcium carbonate precipitation (Hu et al., 2025).

**Table 1:** Mineral composition of test sand (Hu et al., 2025).

Mineral type	CaCO_3_	SiO_2_	Ca_2_ (SO_4_)_2_ (H_2_O)	Others
Mineral content	62.9%	24.5%	7.6%	5%

Note: The data presented in this table are from the same batch of calcareous sand as reported in our previous study (Hu et al., 2025).

**Table 2:** Basic physical properties of test sand (Hu et al., 2025).

Parameters	Coefficient of uniformity	Coefficient of curvature	Specific gravity	Maximum dry density (g/cm^3^)	Minimum dry density (g/cm^3^)	Maximum void ratio	Minimum void ratio
Value	4.484	0.76	2.618	1.702	1.379	0.91	0.54

Note: The data presented in this table are from the same batch of calcareous sand as reported in our previous study (Hu et al., 2025).

**Figure 1:**
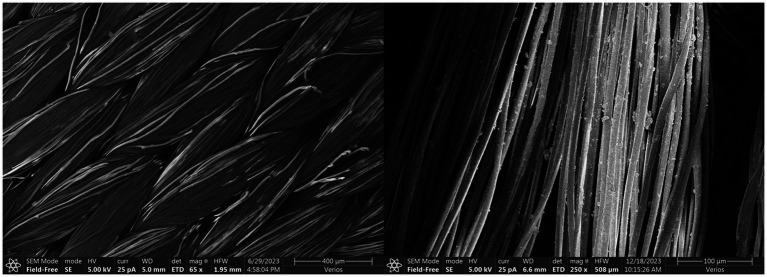
Micro images of hollow fiber membrane.

The fiber membranes used in the tests were PVDF hollow fiber curtain membranes. They have a wall pore size of 0.1 μm, elastic modulus of 450 MPa, and tensile strength of 60 MPa. Scanning electron microscopy (SEM) images reveal the microstructural characteristics of hollow fiber membranes (Figure 1). The fibers are densely arranged and uniformly distributed. This arrangement provides numerous micropores that allow water and other fluids to pass through while effectively retaining larger solid particles or bacteria-with the 0.1 μm wall pores being over an order of magnitude smaller than *Sporosarcina pasteurii* cells (2–3 μm), the membrane wall acts by size exclusion while remaining permeable to the small ionic species involved in MICP. Bacteria therefore accumulate on the membrane outer surface and within the inter-fibre void space rather than penetrating the membrane wall, which allows the fibre surface to act simultaneously as a retention barrier and a localised reservoir for subsequent CaCO₃ precipitation. The uneven surface structure of the fiber membranes increases the adsorption capacity of substances on the fiber surfaces. This improves the bacterial retention rate. These structural characteristics enable hollow fiber membranes to play important roles in bacterial retention and calcium carbonate crystallization promotion during MICP reinforcement processes.

This test selected *Sporosarcina pasteurii* as the bacterial strain for MICP reinforcement, purchased from Guangdong Microbial Culture Collection Center. *Sporosarcina pasteurii* is a classic gram-positive bacterium and an efficient urease-producing microorganism. It exhibiting significantly higher urease activity than most other bacterial species commonly reported in MICP studies. The bacterium presents a characteristic rod-shaped morphology with cell lengths typically ranging from 2–3 μm. It can grow effectively within a temperature range of 15–37 °C. In resource-scarce environments, *Sporosarcina pasteurii* can maintain activity by forming spores. These spores are spherical with diameters of approximately 0.5–1.5 μm and possess extremely strong environmental resistance and long-term survival capabilities.

The culture medium used was yeast extract medium with the following components: yeast extract 20 g/L, ammonium chloride 10 g/L, manganese sulfate 10 mg/L, nickel chloride 24 mg/L, and pH adjusted to 9 using 1 M NaOH solution. Sporosarcina pasteurii was cultivated at a ratio of 1:50 (bacterial solution: culture medium) in a constant temperature shaking incubator at 30 °C and 190 r/min for 18 h. The cultivated bacterial solution had an average urease activity of 1.43 ms/cm/min and an average OD₆₀₀ value of 1.61 (measured after appropriate dilution of the suspension into the linear range of Beer–Lambert’s law, with the reported value back-calculated from the absorbance reading multiplied by the dilution factor). The baseline urease activity and the kinetic effects of pH and urease-inhibitor regulation on this calcareous sand system have been previously characterised by our group in Hu et al. (2025); the present study builds on this biochemical baseline and focuses on the spatial redistribution and macroscopic reinforcement induced by the HFM.

### 2.2 Sample preparation

#### 2.2.1 MICP reinforcement method

This study adopted the one-phase low-pH method for MICP reinforcement. This method adjusts the bacterial solution pH to 5.5. It can effectively delay the bioflocculation time after mixing bacterial solution and nutrient solution. This avoids clogging problems and improves reinforcement uniformity (Hu et al., 2025). The specific reinforcement steps are as follows:

First, 60 mL of deionized water (approximately 1.2 times pore volume) was injected into the specimens through a peristaltic pump at 3 mL/min to remove internal air. Then, 30 mL of successfully cultivated bacterial solution was taken and its pH was reduced to 5.5. It was directly mixed with 90 mL of nutrient solution and stirred uniformly with a glass rod. The 120 mL mixed solution (approximately 2.4 times pore volume) was injected into the specimens using a peristaltic pump at 5 mL/min. Two rounds of reinforcement were performed daily for 8 consecutive days in a 30 °C constant-temperature chamber. After that, the specimens were demolded. The reinforced specimens were soaked in excess deionized water for 24 h to remove soluble salts. Finally, they were dried in a 55 °C oven for 48 h.

#### 2.2.2 Hollow Fiber membrane content and length design

To investigate the effects of hollow fiber membranes on MICP reinforcement effectiveness, this study designed different combinations of hollow fiber membrane contents and lengths. Hollow fiber membrane content (ɱ) was calculated as a percentage of calcareous sand mass. Eight levels were set: 0% (the MICP-only control group, hereafter referred to as the “0% HFM control”), 0.2, 0.4, 0.5, 0.6, 0.7, 0.8, and 1%. All specimens, including the 0% HFM control, received the same MICP treatment protocol described in Section 2.2.1; the only variable across groups is the HFM content and length. Hollow fiber membrane lengths (L) were set at four levels: 5 mm, 10 mm, 15 mm, and 20 mm. Through orthogonal experimental design, 29 groups of specimens with different parameter combinations were prepared. Five parallel specimens were prepared for each group to ensure data reliability.

**Figure 2:**
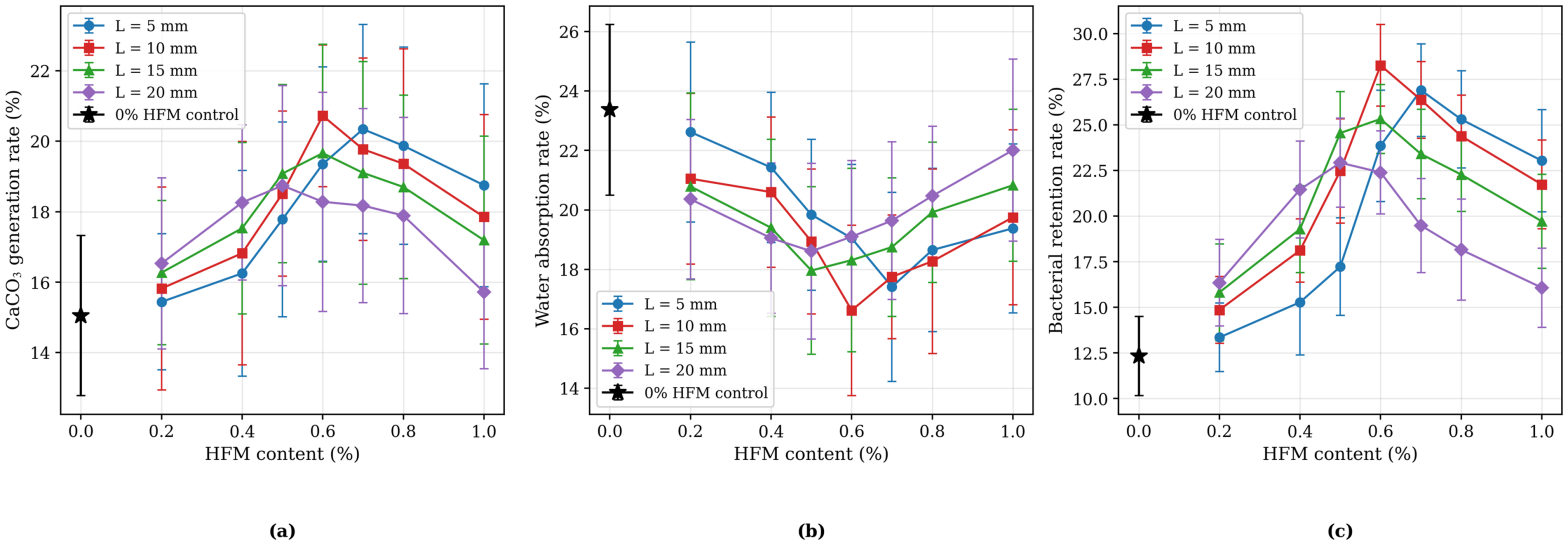
Effects of hollow fiber membrane parameters on physical properties: **(a)** Calcium carbonate generation rate; **(b)** Water absorption rate; **(c)** Bacterial retention rate.

The experimental matrix is therefore designed as a parametric study contrasting MICP-only baseline behavior (0% HFM control) against MICP+HFM behavior, with HFM content and length as the controlled variables. The MICP cementation chemistry adopted here, urease-driven hydrolysis of urea and precipitation of CaCO₃ at 1 mol/L urea + 1 mol/L CaCl₂, has been independently characterised in our earlier work on the same calcareous sand from the South China Sea (Hu et al., 2025), which provides a previously published baseline for the MICP-only response in this material system. Additional reference groups, for example, abiotic specimens containing HFM but no bacteria, specimens with HFM and inactivated bacterial suspension, and specimens reinforced with solid (non-hollow) fibres of the same diameter, were not included in the present study because the focus here is the parametric optimisation of HFM geometry and content under an active MICP scheme. These reference groups would help to isolate the purely physical contribution of the HFM, the abiotic chemical contribution of the cementation solution, and the role of the hollow geometry, and are identified as a priority for follow-up work. For each of the 29 orthogonal configurations, five parallel specimens were prepared and tested. Configuration-level means were retained for all responses; in addition, sample standard deviations of the three physical responses (CaCO₃ generation rate, water absorption rate, and bacterial retention rate) were preserved and are used to construct the error bars in Figure 2. Two-way analysis of variance (ANOVA) and orthogonal range analysis (R analysis) were performed at the configuration level for each response variable, with HFM content (seven levels: 0.2–1.0%) and HFM length (four levels: 5–20 mm) as factors.

#### 2.2.3 Specimen preparation

**Figure 3:**
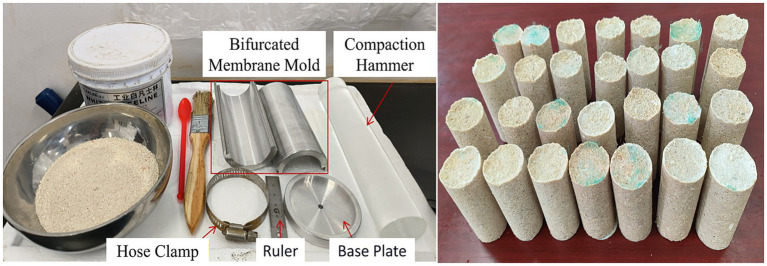
Specimen preparation tools and reinforced specimens.

Specimens were prepared using the wet sand layered compaction method. Cylindrical specimens with dimensions of φ39.1 mm × 80 mm were prepared using custom stainless-steel molds, as shown in Figure 3. The specific preparation steps are as follows:

The split mold was assembled using hose clamps and tightly connected to the perforated base. Vaseline was applied to the inner walls of the mold and PVC film was placed to facilitate demolding. Cut cleaning cloth was placed at the bottom of the mold to prevent sand loss. 128 g of calcareous sand was weighed and mixed with hollow fiber membranes of corresponding proportions and lengths according to the test scheme. For specimens with added fiber membranes, the fiber membranes were first cut to designed lengths, then thoroughly mixed with calcareous sand to ensure uniform distribution of fiber membranes in the sand. 8 g of deionized water (approximately 5% of calcareous sand mass) was added and mixed uniformly. The mixture was placed into the mold in three layers. After each layer was filled, it was uniformly compacted using a compactor. The interfaces between layers were lightly brushed to prevent obvious layering in the specimens. After compacting the specimens to 80 mm height, cleaning cloth was placed on top to prevent liquid erosion of the specimen surface during reinforcement, which could cause surface irregularities.

The distribution of hollow fiber membranes in specimens has important effects on reinforcement effectiveness. Under low content conditions, fiber membranes can form effective spatial network structures between sand particles. Under high content conditions, special attention must be paid to preventing fiber membrane aggregation and entanglement to ensure uniform distribution in the specimens.

### 2.3 Test methods

#### 2.3.1 Physical property tests

Physical property tests include calcium carbonate generation rate, water absorption rate, and bacterial retention rate tests. These tests evaluate the effects of hollow fiber membranes on the MICP reinforcement process.

Calcium carbonate generation rate testing was conducted using the weighing method. First, the mass of specimens to be reinforced ( $M_{1}$ ) was measured. After MICP reinforcement, specimens were soaked in deionized water tanks for 24 h to remove soluble salts. Then they were dried in a constant temperature oven at 55 °C for 48 h and weighed again ( $M_{2}$ ). The calcium carbonate generation rate ( $M_{\mathrm{ca}}$ ) was calculated using the Equation 1:


$$M_{\mathrm{ca}}=\frac{M_{2}-M_{1}}{M_{1}}\times 100\%$$ (1)

This method avoids the destruction of calcium carbonate components in calcareous sand itself caused by acid washing methods. It can accurately determine the calcium carbonate content generated during the MICP process.

Bacterial retention rate testing was used to evaluate the bacterial retention capability of hollow fiber membranes. To eliminate the influence of cementing solution, the conventional reinforcement method was adopted and only the first bacterial injection process was recorded. The OD_600_ value of the bacterial solution was measured before injection through the peristaltic pump and recorded as $O_{1}$ . After bacterial injection, the OD_600_ value of the bacterial solution flowing out from the bottom of the specimen was measured and recorded as $O_{2}$ . The bacterial retention rate was calculated using the Equation 2:


$$O=\frac{O_{1}-O_{2}}{O_{1}}\times 100\%$$ (2)

By definition, O reflects the system-level retention of the entire specimen (HFM-modified sand column). The specific contribution of the HFM, decoupled from the bare calcareous sand matrix, is therefore obtained by comparing the HFM-containing specimens with the 0% HFM control group.

#### 2.3.2 Mechanical property tests

**Figure 4:**
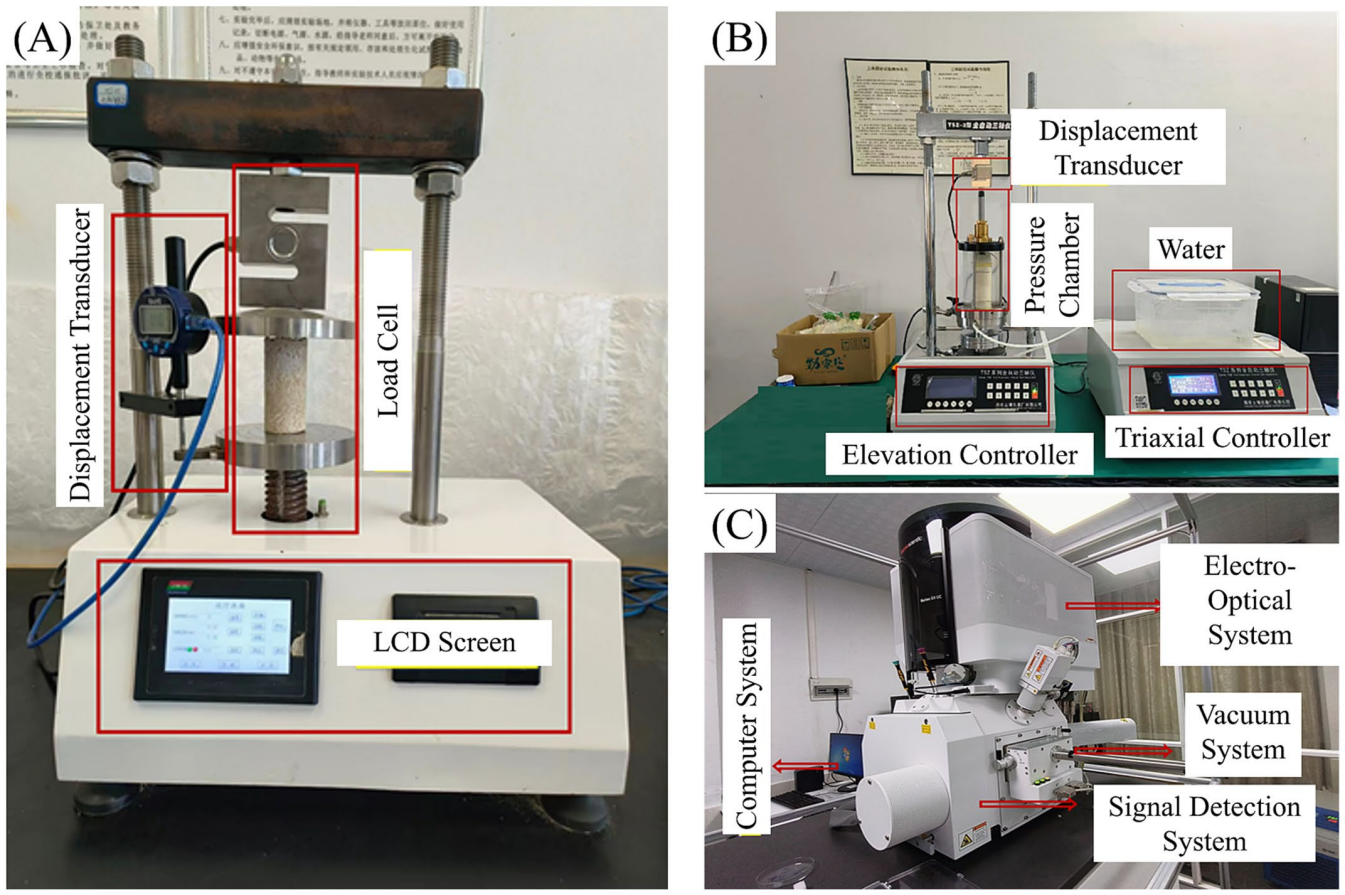
Test equipment. **(A)** UCS testing machine; **(B)** UU triaxial apparatus; **(C)** SEM.

Unconfined compressive strength (UCS) tests were conducted using an automatic compression testing machine developed by Zhejiang Geo-Technology Co., Ltd. (Figure 4A). The loading rate was set to 1 mm/min during testing. The reinforced specimens were placed at the center of the pressure plate. Loading began after balancing the self-weight and continued until either the axial strain reached 15% or the post-peak stress dropped to 85% of the peak value, whichever occurred first. Stress–strain curves were recorded in real-time during the test process to obtain mechanical parameters such as peak strength and elastic modulus.

Unconsolidated undrained shear tests (UU) were conducted using a TSZ series automatic triaxial apparatus from Nanjing Soil Instrument Factory Co., Ltd. (Figure 4B). Specimens had a height of 80 mm and diameter of 39.1 mm. Three confining pressure levels were set: 100 kPa, 200 kPa, and 300 kPa. The shear rate was set to 0.8 mm/min, and tests were terminated when axial strain reached 15%. Stress–strain curves under different confining pressures were obtained through testing. Shear strength parameters such as cohesion c and internal friction angle *φ* were calculated according to Mohr-Coulomb strength theory. The specimens entered the UU stage directly after the 55 °C oven-drying step of the MICP post-treatment protocol and were not subjected to back-pressure saturation prior to shearing; the strengths reported here therefore characterise the shear response of the reinforced specimens in their post-cementation (quasi-dry) state and should not be directly compared with strengths obtained under fully saturated conditions.

#### 2.3.3 Microstructural analysis

Microstructural observation of reinforced specimens was conducted using a Verios 5 XHR SEM ultra-high resolution scanning electron microscope produced by Thermo Fisher Scientific (Figure 4C). Failed specimens were selected and cut to fingernail size for drying treatment. Due to the poor conductivity of calcareous sand, samples were firmly attached to metal sample stages using conductive adhesive and subjected to gold coating treatment. The treated samples were placed in the scanning electron microscope vacuum chamber. Photography was conducted under pressure after meeting vacuum requirements. SEM was used to observe calcium carbonate crystal morphology, cementation conditions between hollow fiber membranes and sand particles, and distribution characteristics of fiber membranes in specimens. The reinforcement mechanisms of MICP combined with hollow fiber membranes were analyzed from a microscopic perspective.

## 3 Results and analysis

### 3.1 Physical property analysis

Hollow fiber membranes primarily influence MICP reinforcement through modifications in physical properties. Figure 2 illustrates the interactive effects of fiber membrane length and content on three critical physical parameters: (a) calcium carbonate generation rate indicates MICP reaction efficiency and precipitation quantity; (b) water absorption rate characterizes the extent of pore filling within specimens; (c) bacterial retention rate demonstrates the bacterial capture capacity of fiber membranes. These parameters collectively reveal the reinforcement mechanisms from different perspectives. The trend lines provide insights into optimal fiber membrane configurations. The 0% HFM control specimens exhibited a calcium carbonate generation rate of 15.05%, water absorption rate of 23.37%, and bacterial retention rate of 12.33%. In particular, the bacterial retention rate of 12.33% in the 0% HFM control quantifies the baseline filtration and adsorption capacity of the calcareous sand matrix itself. Subtracting this baseline from the 28.27% retention obtained at the optimal HFM configuration (0.6%, 10 mm) yields a net HFM-attributable increase of 15.94 percentage points (a 2.29-fold relative enhancement). This quantitative comparison successfully decouples the specific contribution of the HFMs from the background sand skeleton. The data in Figure 2 demonstrate that fiber membrane incorporation significantly enhances specimen treatment effectiveness.

The calcium carbonate generation rate curves (Figure 2a) exhibit a characteristic unimodal trend. The peak values (20.06–20.72%) are concentrated at fiber membrane contents of 0.55–0.7% and lengths of 8–12 mm, with the maximum value of 20.72% occurring near 0.6% content and 10 mm length. This high-value zone indicates that optimal fiber membrane dosages effectively enhance MICP reactions. At contents below 0.4%, calcium carbonate generation rates remain consistently below 17.5%, suggesting insufficient fiber membranes to provide adequate bacterial attachment sites. Conversely, when contents exceed 0.8% or lengths exceed 15 mm, generation rates decline below 18% due to fiber membrane agglomeration, which occupies reaction space and impedes uniform nutrient distribution. The steeper variation along the content axis compared with the length axis demonstrates that calcium carbonate generation rates are more sensitive to content variations than to length changes.

The water absorption trends (Figure 2b) demonstrates a distinct negative correlation with calcium carbonate generation rates. The minimum water absorption values (16.62–17.37%) coincides with the high calcium carbonate generation region at contents of 0.5–0.7% and lengths of 8–12 mm. This correspondence directly confirms effective pore filling by calcium carbonate precipitation. Notably, both low content-short fiber regions (e.g., 0.2%, 5 mm) and high content-long fiber regions (e.g., 1.0%, 20 mm) exhibit water absorption rates exceeding 20%, approaching or even surpassing that of the 0% HFM control (23.37%). A localized peak (>22%) appears near 0.2–0.3% content and 5 mm length, indicating that fiber membrane addition under these conditions not only fails to promote pore filling but may increase porosity by disrupting sand structure. The relatively narrow spread of the four length-curves within the optimal content range suggests that moderate adjustments in fiber membrane length have relatively minor effects on water absorption within the optimal content range.

The bacterial retention rate distribution (Figure 2c) follows a single-peak pattern with the maximum located at intermediate fiber content. The high-value region (>25%) is concentrated at 0.6–0.7% content and 5–15 mm length, while the low-value region (<16%) is found uniformly at 0.2% content across all lengths and at 1.0% content with 20 mm length. The optimal point coincides with that for calcium carbonate generation, at 0.6% content and 10 mm length, where bacterial retention reaches 28.27%—corresponding to a 2.29-fold increase over the 0% HFM control (12.33%). This single-peak distribution is consistent with a dominant size-exclusion retention mechanism: increasing fiber content augments the available membrane surface for bacterial attachment, but excessive content (≥0.8%) leads to fiber agglomeration and reduced effective retention area, while excessive length (20 mm) at high content promotes fiber bending and overlap. The position of the bacterial retention optimum at the same configuration as the CaCO₃ optimum is consistent with a coupled retention–mineralisation pathway, in which retained bacteria concentrate urease activity locally and drive carbonate precipitation in the immediate vicinity of the membrane.

Integrating the three physical parameter distributions identifies the optimal configuration zone at fiber membrane contents of 0.5%–0.7% and lengths of 8–12 mm, with 0.6% content and 10 mm length representing the ideal combination. Under these conditions, calcium carbonate generation rate reaches 20.72% (37.7% increase), water absorption decreases to 16.62% (28.9% reduction), and bacterial retention rate achieves 28.27% (2.29 × enhancement). This synergistic optimization demonstrates that hollow fiber membranes enhance bacterial retention efficiency, promote calcium carbonate precipitation, and effectively fill sand pores, resulting in significant MICP reinforcement improvement.

Two-way ANOVA on the 28-configuration matrix indicates that HFM content is the dominant factor for the physical responses, with significant main effects on CaCO₃ generation rate (*F* = 8.43, *p* < 0.001), water absorption rate (*F* = 4.61, *p* = 0.005), and bacterial retention rate (*F* = 6.06, *p* = 0.001). The main effect of HFM length is not statistically significant for any physical response (*p* > 0.08), although the interaction with content drives the empirical optimum to 10 mm. Orthogonal range analysis confirms this hierarchy: R(HFM content) is significantly larger than R(HFM length), and the optimal level converges on 0.6% content and 10 mm length. Relative to the 0% HFM control, this optimal configuration produces a 37.7% increase in CaCO₃ generation rate, a 28.9% reduction in water absorption rate, and a 2.29-fold increase in bacterial retention rate.

### 3.2 Unconfined compressive behavior analysis

#### 3.2.1 Stress–strain response and peak strength

**Figure 5:**
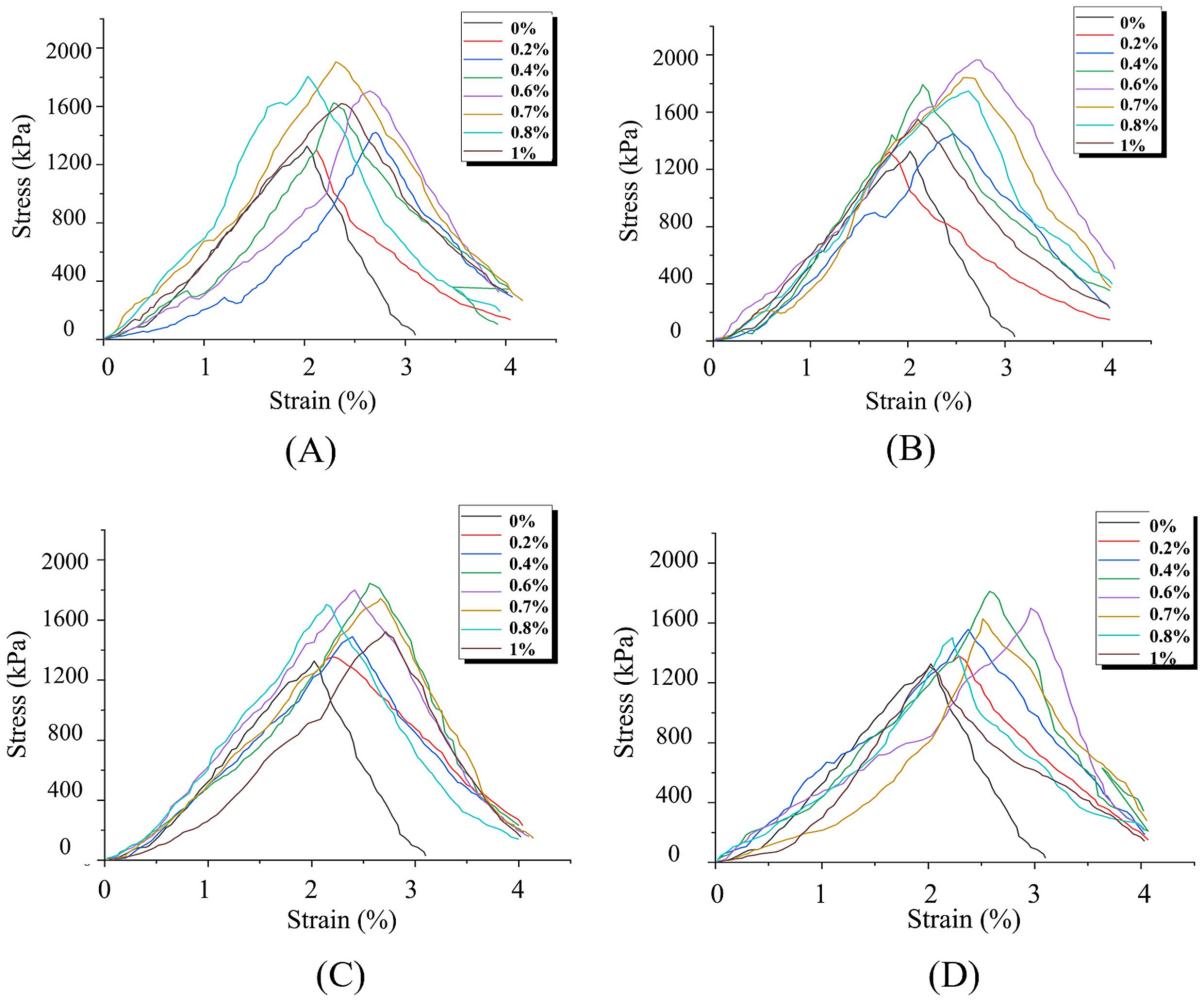
UCS curves under different hollow fiber membrane contents and lengths: **(A)** *L* = 5 mm; **(B)** *L* = 10 mm; **(C)** *L* = 15 mm; **(D)** *L* = 20 mm.

Figure 5 presents the stress–strain curves of unconfined compression test specimens under different hollow fiber membrane contents and lengths.

Figure 5 demonstrates that regardless of hollow fiber membrane content and length variations, all specimens exhibit similar stress–strain behavior characterized by three distinct phases:

Elastic phase: Under initial vertical loading, stress increases linearly with strain. Hollow fiber membranes effectively retain bacteria, facilitating bacterial attachment between fiber membranes and calcareous sand particles. This generates calcium carbonate crystals with cementing capabilities, forming a robust composite structure that provides specimens with enhanced initial strength and toughness.

Damage phase: As strain increases, microcracks initiate at weak cementation zones within specimens. Fiber membranes near microcracks experience tensile stress from surrounding soil movement, partially restraining crack propagation. Continued loading causes gradual crack extension and formation of localized failure planes.

Failure phase: Sustained loading leads to peak stress, followed by rapid stress reduction. Specimens fracture along localized failure planes; however, fiber membrane presence delays failure progression, enabling specimens to maintain residual compressive capacity during initial failure until fiber membrane bridging effects cease, resulting in complete failure.

**Figure 6:**
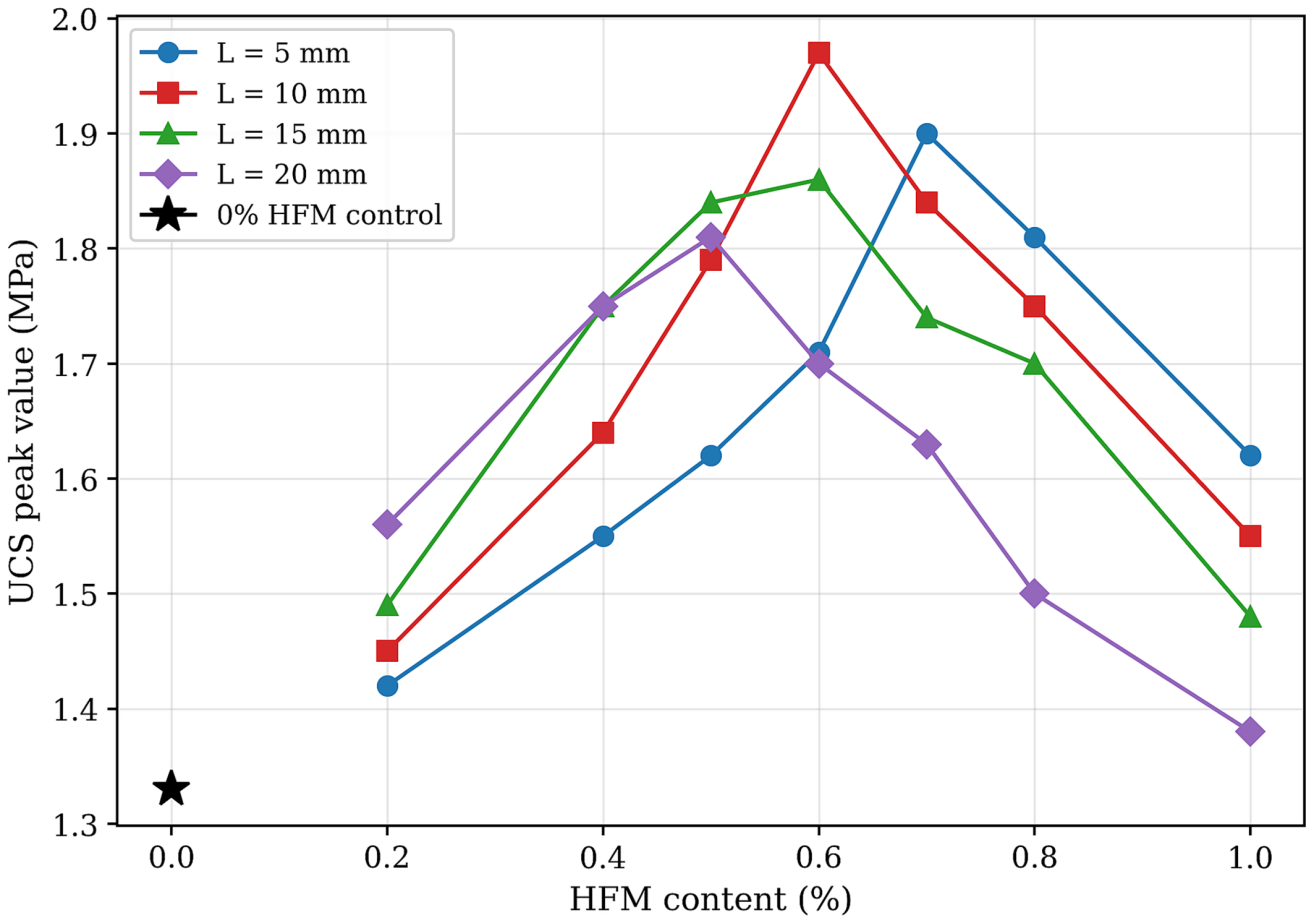
UCS peak strength under different hollow fiber membrane contents and lengths.

Figure 6 illustrates the distribution of UCS peak values for MICP-treated calcareous sand under various hollow fiber membrane configurations.

Figure 6 demonstrates that hollow fiber membrane incorporation significantly enhances specimen UCS, with values following an initial increase followed by decrease with increasing fiber membrane content. The 0% HFM control exhibits a UCS of 1.33 MPa. At 5 mm fiber length, specimens achieve maximum strength of 1.90 MPa at 0.7% content, representing a 1.44-fold increase over the 0% HFM control. For 10 mm fiber length, peak strength reaches 1.97 MPa at 0.6% content (1.48-fold increase), constituting the highest value among all test groups. Specimens with 15 mm fibers attain 1.86 MPa at 0.6% content (40% enhancement), while 20 mm fibers achieve 1.81 MPa at 0.5% content (36% improvement).

Experimental data further reveal that UCS, calcium carbonate generation rate, and bacterial retention rate exhibit similar trends with varying hollow fiber membrane content. This confirms that appropriate fiber membrane addition effectively enhances bacterial retention, leading to increased calcium carbonate precipitation within specimens. Enhanced bacterial retention promotes formation of stable “calcareous sand–calcium carbonate crystal–hollow fiber membrane” cemented matrices, resulting in significant UCS improvements. Statistical analysis corroborates these macroscopic findings; two-way ANOVA shows a significant main effect of HFM content on UCS peak value (*F* = 5.88, *p* = 0.0015). Range analysis further confirms the optimum at 0.6% content and 10 mm length, yielding a 48.1% increase (1.33 to 1.97 MPa) over the 0% HFM control.

#### 3.2.2 Failure mode analysis

**Figure 7:**
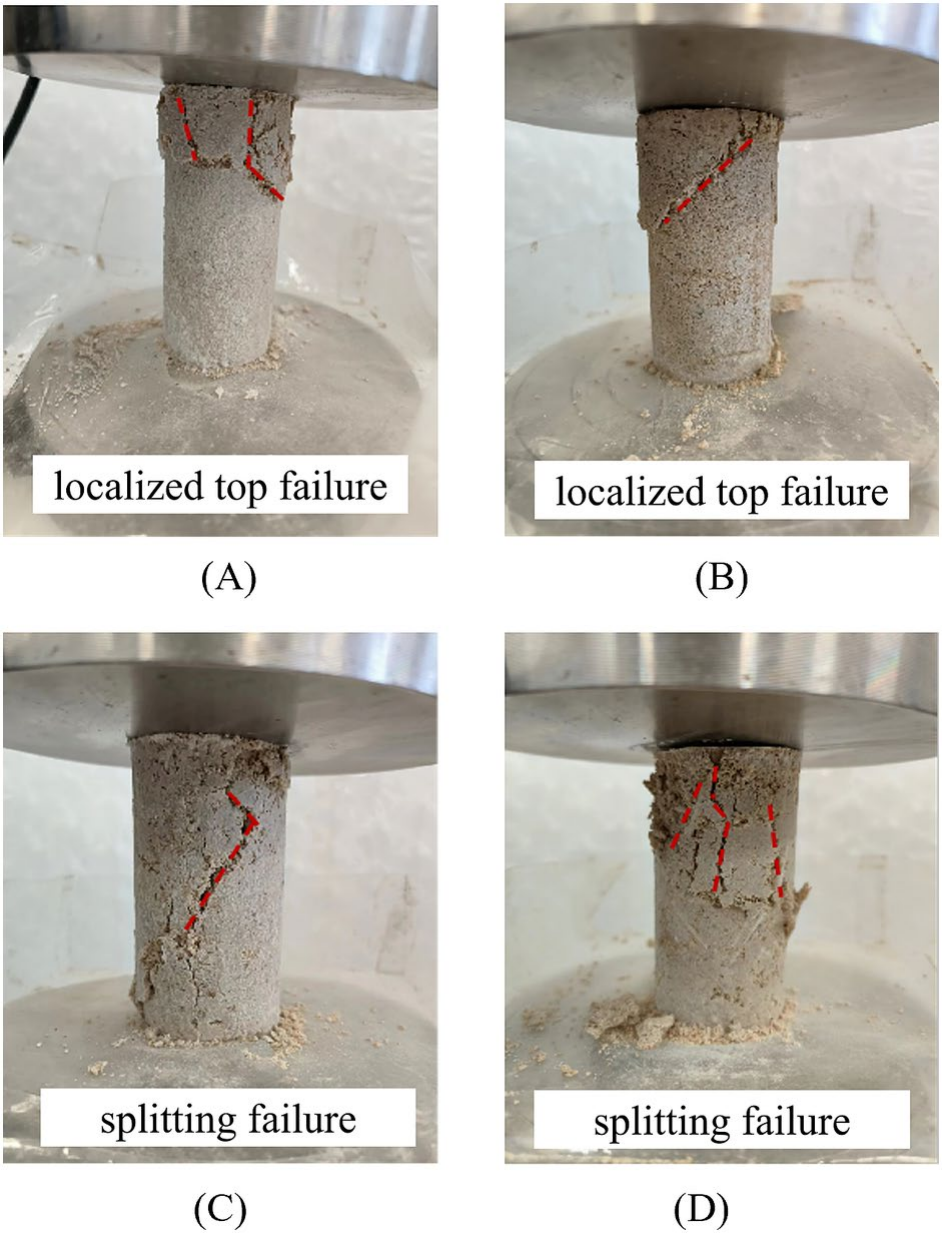
Unconfined compression failure modes: **(A)** ɱ = 0%, *L* = 0 mm; **(B)** ɱ = 0.5%, *L* = 15 mm; **(C)** ɱ = 0.6%, *L* = 10 mm; **(D)** ɱ = 1%, *L* = 20 mm.

The failure modes in unconfined compression tests reflect the influence of hollow fiber membranes on soil structural integrity. Figure 7 presents typical failure patterns of specimens under different fiber membrane contents and lengths.

Figure 7 reveals systematic evolution of failure modes with varying fiber membrane content and length. The 0% HFM control specimens (Figure 7A) and low-content specimens (0.2–0.4%) primarily exhibit localized top failure, with damage concentrated within the upper third of specimen height, demonstrating typical crushing failure characteristics. This occurs because insufficient fiber membranes fail to establish effective spatial constraint networks, resulting in stress concentration and localized crushing under axial loading.

When fiber membrane content increases to 0.5–0.6% with lengths of 10–15 mm (Figures 7B,C), failure modes transition to penetrative splitting failure. Primary cracks propagate from top to bottom at inclination angles of approximately 45°–60°. This transition indicates that optimal fiber membrane dosages create effective three-dimensional network structures, promoting more uniform stress distribution. When weak planes begin yielding, fiber membrane bridging effects guide crack propagation along specific paths, forming single dominant failure planes. Under this failure mode, specimens exhibit enhanced peak strength and improved structural integrity.

At high contents (0.7%–1%), particularly with long fiber membranes (15–20 mm) (Figure 7D), failure modes display composite characteristics combining localized top crushing with multiple inclined cracks. This complex failure pattern stems from non-uniform fiber membrane distribution—excessive fiber membranes tend to agglomerate locally, creating heterogeneous strength distribution and multiple potential failure planes. During failure, specimens develop simultaneously along multiple crack surfaces, paradoxically reducing overall structural integrity.

### 3.3 Unconsolidated undrained shear strength characteristics

#### 3.3.1 Peak strength

**Figure 8:**
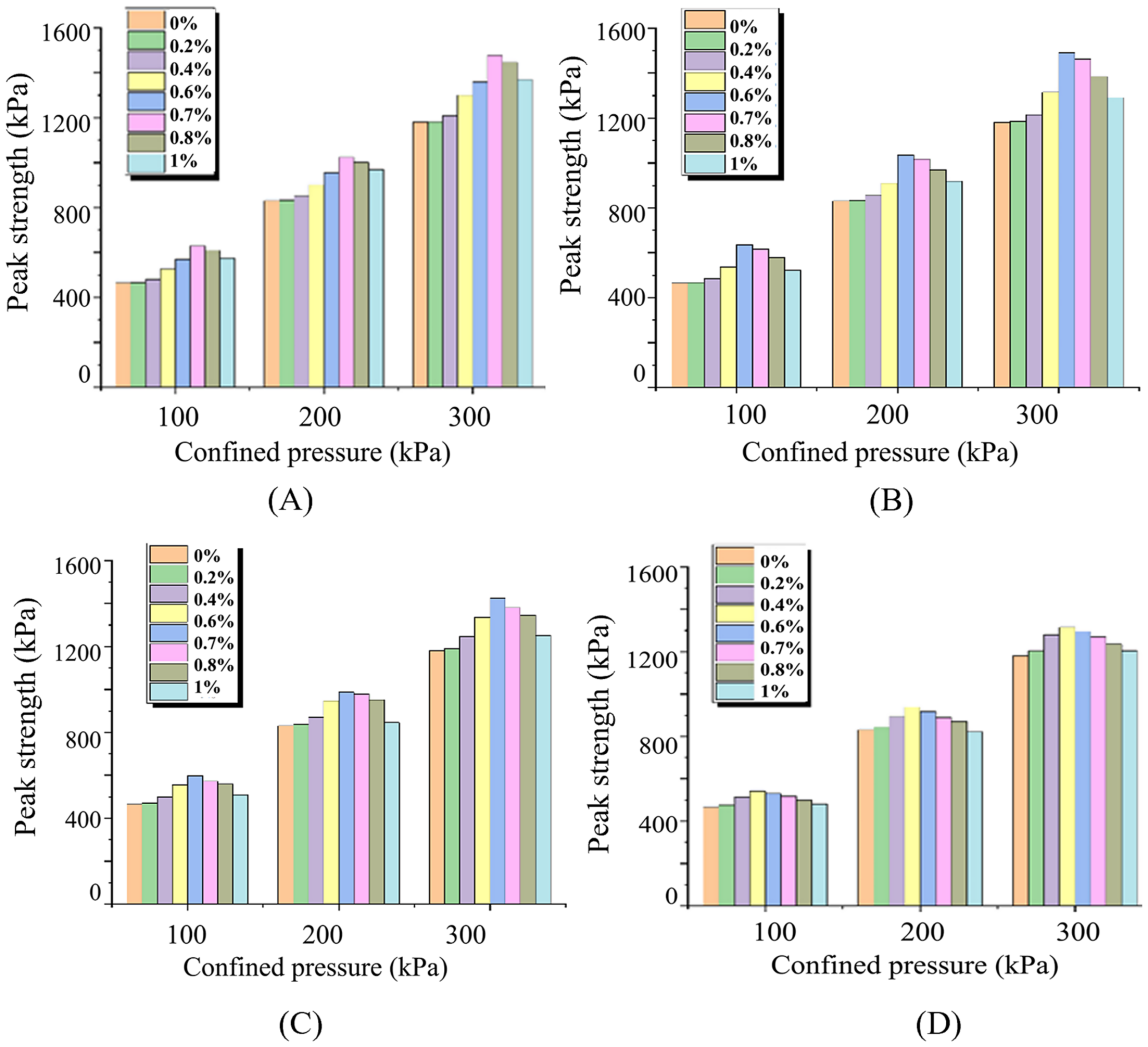
Effects of hollow fiber membrane content and length on UU test peak strength: **(A)** *L* = 5 mm; **(B)** *L* = 10 mm; **(C)** *L* = 15 mm; **(D)** *L* = 20 mm.

UU tests were conducted on specimens with varying hollow fiber membrane parameters. Figure 8 presents peak strengths from UU tests under different hollow fiber membrane contents and lengths. Results demonstrate that regardless of confining pressure and hollow fiber membrane length variations, specimens with hollow fiber membranes consistently exhibit higher peak strengths than the 0% HFM control across all confining pressures. Peak strength follows an initial increase followed by decrease with increasing hollow fiber membrane content.

Under optimal conditions (0.6% content, 10 mm length), specimens achieved peak strengths of 635.02 kPa, 1034.18 kPa, and 1490.30 kPa at confining pressures of 100 kPa, 200 kPa, and 300 kPa, respectively. These values represent improvements of 36.64, 24.54, and 26.35% compared to the 0% HFM control.

#### 3.3.2 Cohesion and internal friction angle

**Figure 9:**
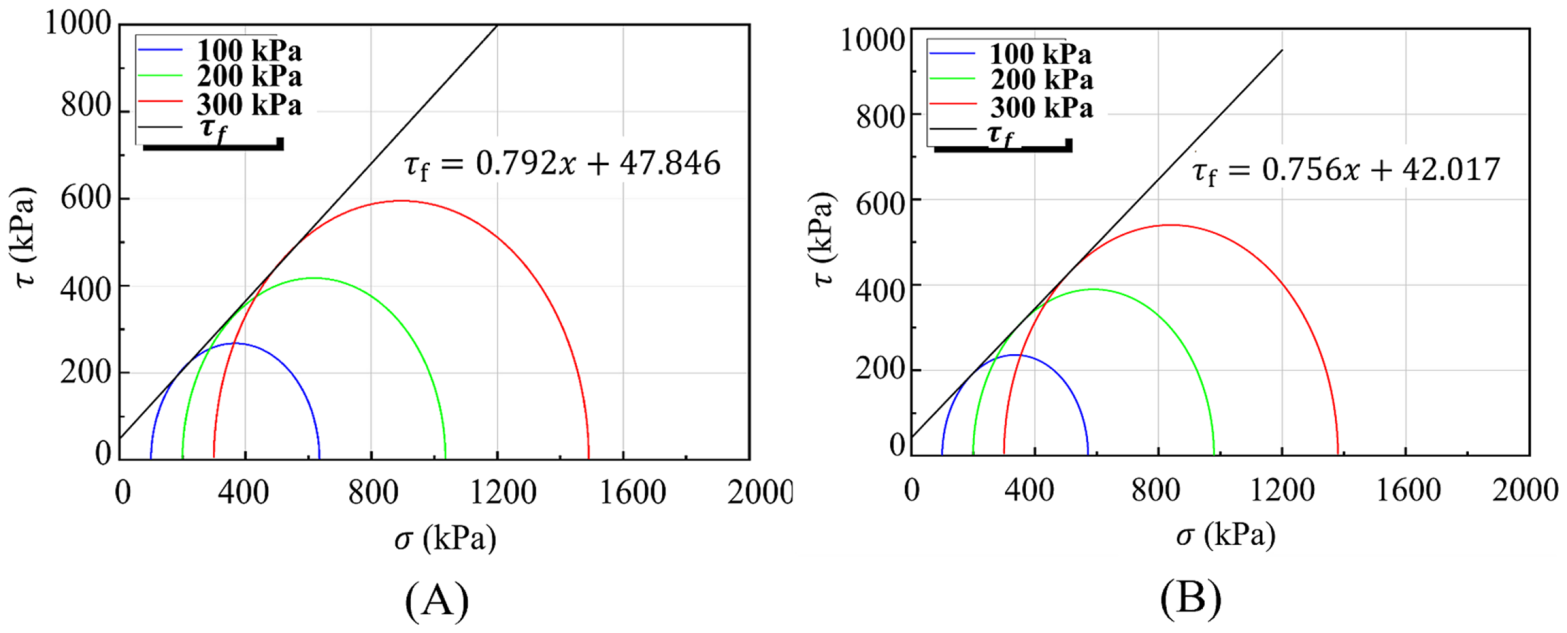
Strength envelope of treated calcareous sand: **(A)** ɱ = 0.6%, *L* = 10 mm; **(B)** ɱ = 0.7%, *L* = 15 mm.

**Figure 10:**
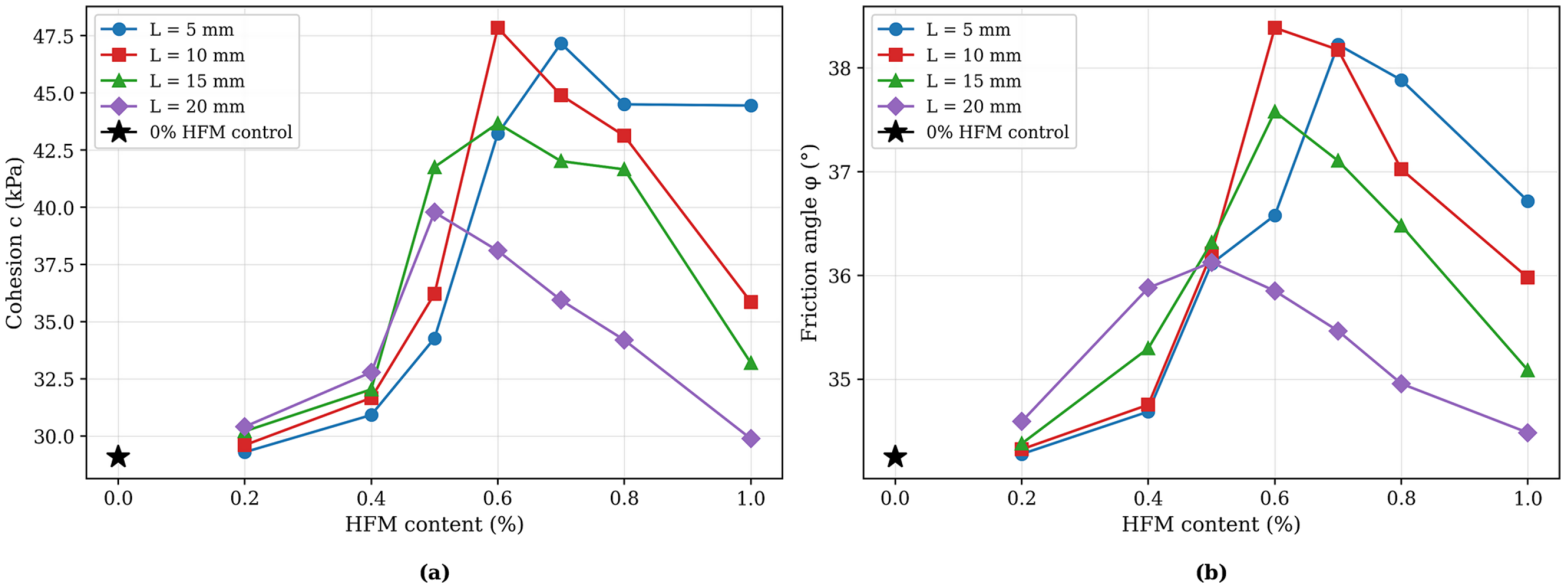
Relationship between hollow fiber membrane parameters and shear strength indices: **(a)** Cohesion and **(b)** internal friction angle.

To further investigate the effects of hollow fiber membranes on specimen shear strength, peak strengths under various confining pressures were designated as $\sigma_{1}$ , while test confining pressures (100 kPa, 200 kPa, 300 kPa) were designated as $\sigma_{3}$ . Mohr stress circles and corresponding strength envelopes were constructed with centers at $(\sigma_{1}+\sigma_{3})/2$ and radii of $(\sigma_{1}-\sigma_{3})/2$ , as shown in Figure 9. Cohesion (c) and internal friction angle (*φ*) were calculated for each content level, with results presented in Figure 10.

Figure 10 demonstrates that hollow fiber membranes enhance both cohesion and internal friction angle, though the magnitude of improvement differs significantly. Cohesion exhibits an initial increase followed by decrease with increasing hollow fiber membrane content. The 0% HFM control shows cohesion of 29.087 kPa and internal friction angle of 34.251°. Under optimal conditions (0.6% content, 10 mm length), cohesion reaches 47.846 kPa—a 1.65-fold increase representing 64.49% enhancement over the 0% HFM control. Internal friction angle achieves 38.386°, corresponding to only 12.07% improvement.

These findings indicate that hollow fiber membranes significantly influence shear strength characteristics of calcareous sand specimens, particularly cohesion enhancement. While hollow fiber membranes show limited effectiveness in improving internal friction angle, overall shear strength enhancement occurs primarily through cohesion increases. This substantial cohesion improvement results from cementation effects of MICP-generated calcium carbonate between sand particles, combined with spatial constraint effects from hollow fiber membrane networks.

Two-way ANOVA confirms the significant main effect of HFM content on both cohesion c (*F* = 7.94, *p* < 0.001) and friction angle φ (*F* = 6.57, *p* < 0.001). The optimal 0.6%/10 mm configuration yields a 64.5% increase in cohesion c (29.09 to 47.85 kPa) and a 12.1% increase in friction angle φ (34.25° to 38.39°).

#### 3.3.3 Failure mode analysis

**Figure 11:**
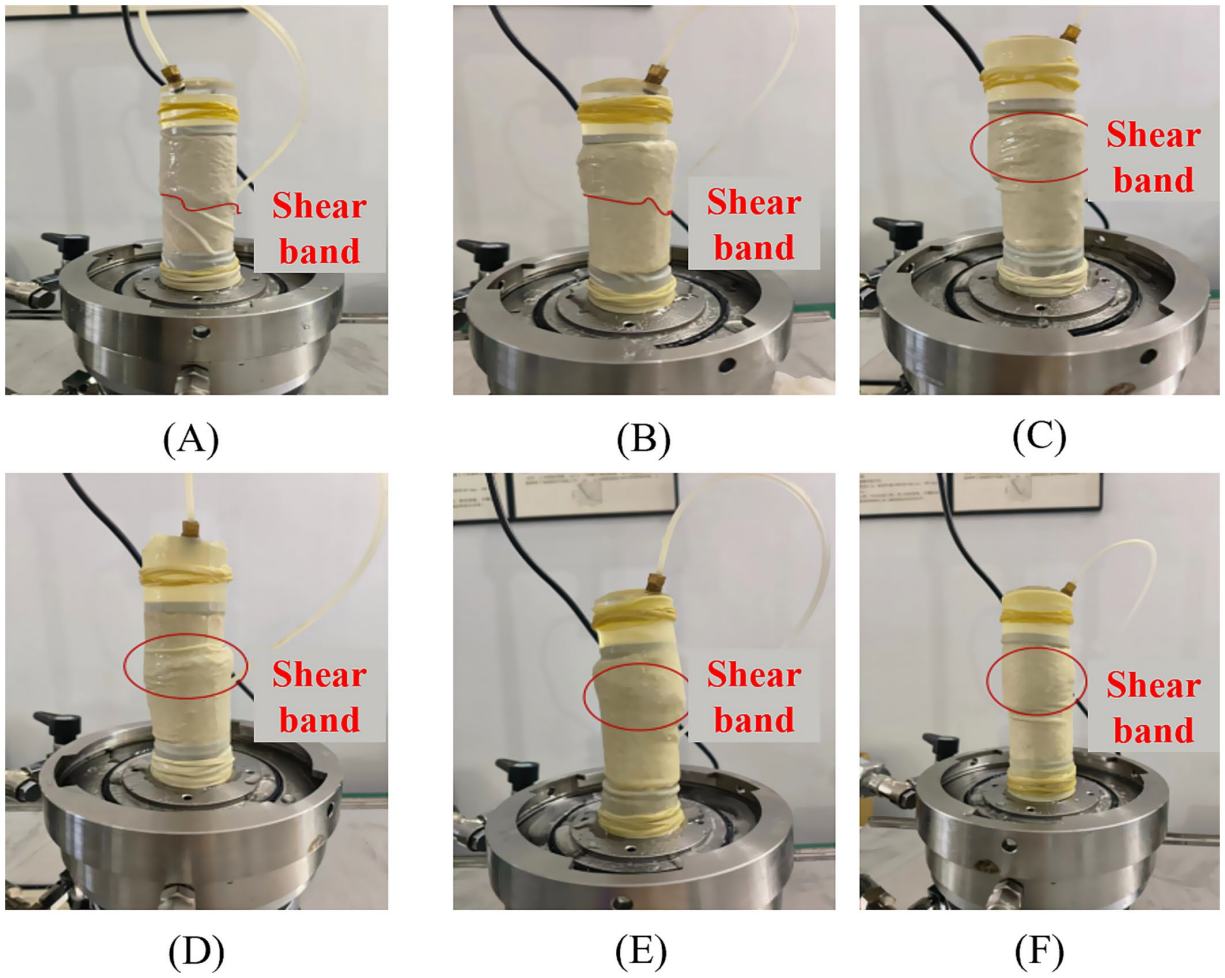
UU test failure modes under different hollow fiber membrane configurations: **(A)** 100 kPa, ɱ = 0%, *L* = 0 mm; **(B)** 200 kPa, ɱ = 0.4%, *L* = 20 mm; **(C)** 200 kPa, ɱ = 0.5%, *L* = 15 mm; **(D)** 300 kPa, ɱ = 0.6%, *L* = 10 mm; **(E)** 100 kPa, ɱ = 0.7%, *L* = 5 mm; **(F)** 300 kPa, ɱ = 1%, *L* = 5 mm.

Failure modes in UU tests reflect the influence of confining pressure on reinforced specimen failure mechanisms. Figure 11 presents failure patterns of specimens under representative confining pressures and hollow fiber membrane configurations.

Figure 11 shows that the 0% HFM control specimens and low hollow fiber membrane content specimens predominantly fail through central shear cracking. However, as hollow fiber membrane content increases, failure modes gradually shift toward top-dominated shear failure. This evolution indicates that hollow fiber membranes significantly enhance cementation between loose sand particles, enabling soil consolidation into more homogeneous structures. Nevertheless, hollow fiber membrane-reinforced specimens still exhibit brittle failure characteristics, suggesting that while hollow fiber membranes improve overall structural stability, their capacity for enhancing ductility remains limited. Therefore, hollow fiber membrane performance in terms of ductility enhancement and brittle failure resistance requires further optimization.

### 3.4 Microscopic mechanism analysis

**Figure 12:**
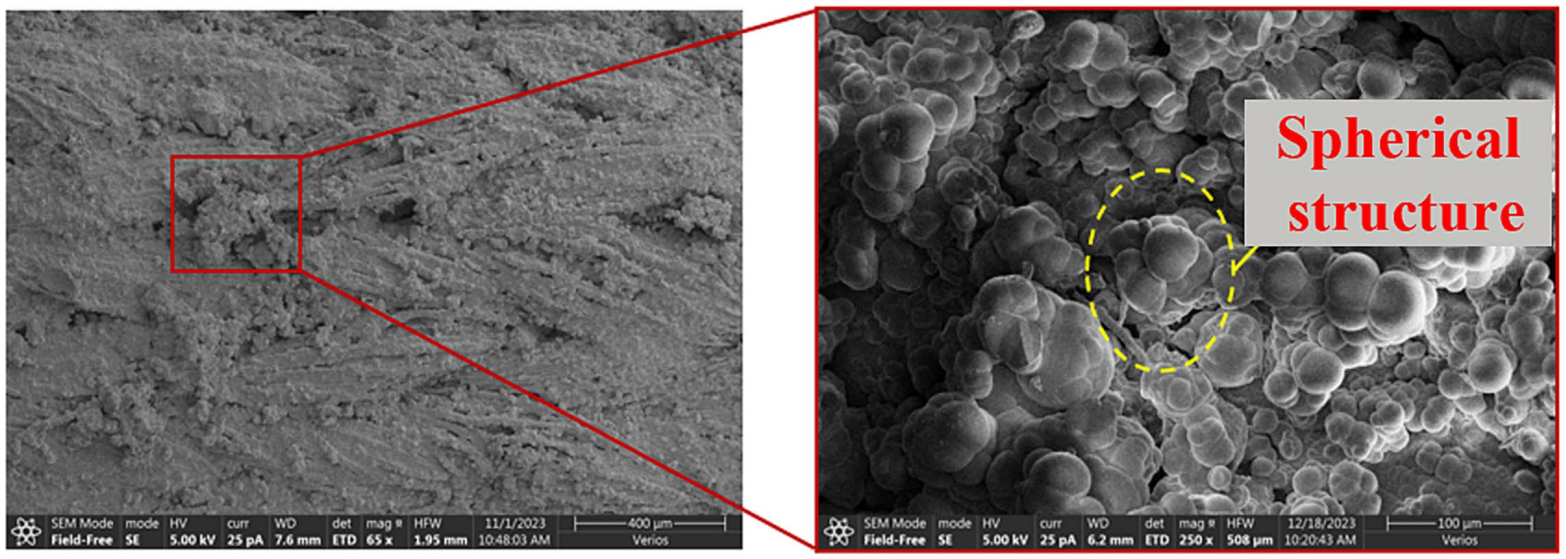
Bacterial retention and calcium carbonate precipitation on hollow fiber membranes.

**Figure 13:**
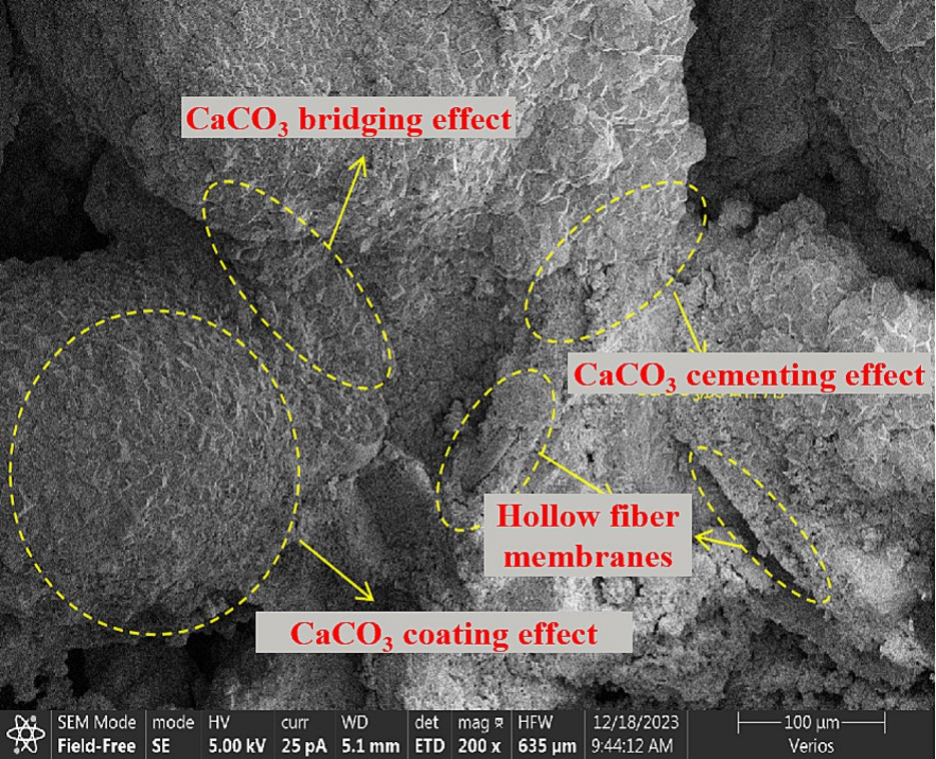
Network structure of hollow fiber membrane-sand composites.

Figures 12, 13 present SEM microscopic results of calcareous sand treated by MICP combined with hollow fiber membranes.

Figure 12 demonstrates that hollow fiber membranes effectively retain bacteria, enabling bacterial reaction with cementing solution to generate abundant calcium carbonate crystals on hollow fiber membrane surfaces. The microporous structure of hollow fiber membranes (0.1 μm pore size) provides a high-roughness outer surface that favours bacterial attachment and subsequent colonisation, facilitating bacterial colonization and MICP reactions on membrane surfaces. Observations reveal extensive calcium carbonate crystal coverage on hollow fiber membranes, forming dense crystalline layers. These crystalline layers not only increase membrane surface roughness but also enhance interlocking forces and frictional cementation area with sand particles. Bacterial retention rate data further validate this mechanism—under optimal conditions, bacterial retention reaches 28.27%, representing a 2.29-fold increase over the 0% HFM control. This significant enhancement indicates that hollow fiber membrane structures simultaneously retain bacteria while providing favorable growth environments, promoting MICP reactions.

Figure 13 reveals that MICP reinforcement mechanisms in sand can be categorized into three primary actions: bridging, cementation, and coating. Bridging action occurs when calcium carbonate crystals precipitate and aggregate between adjacent but non-contacting calcareous sand particles, serving as supporting media to establish contact through crystal bridges. Cementation action involves calcium carbonate crystal deposition around sand particle contact points, strengthening inter-particle contacts and reducing relative sliding potential. Coating action results from continuous calcium carbonate crystal accumulation on calcareous sand surfaces, filling particle pores and forming calcium carbonate shells that increase particle surface area and inter-particle friction.

However, at high hollow fiber membrane contents and lengths, limited specimen space causes non-uniform hollow fiber membrane distribution, resulting in bending, agglomeration, and overlap phenomena. Excessive and overly long hollow fiber membranes reduce effective bacterial survival space within specimens, hindering calcium carbonate crystal formation and distribution. This explains why optimal reinforcement effects occur at 0.6% hollow fiber membrane content and 10 mm length—this configuration achieves optimal balance among hollow fiber membrane distribution, bacterial retention, and calcium carbonate generation.

**Figure 14:**
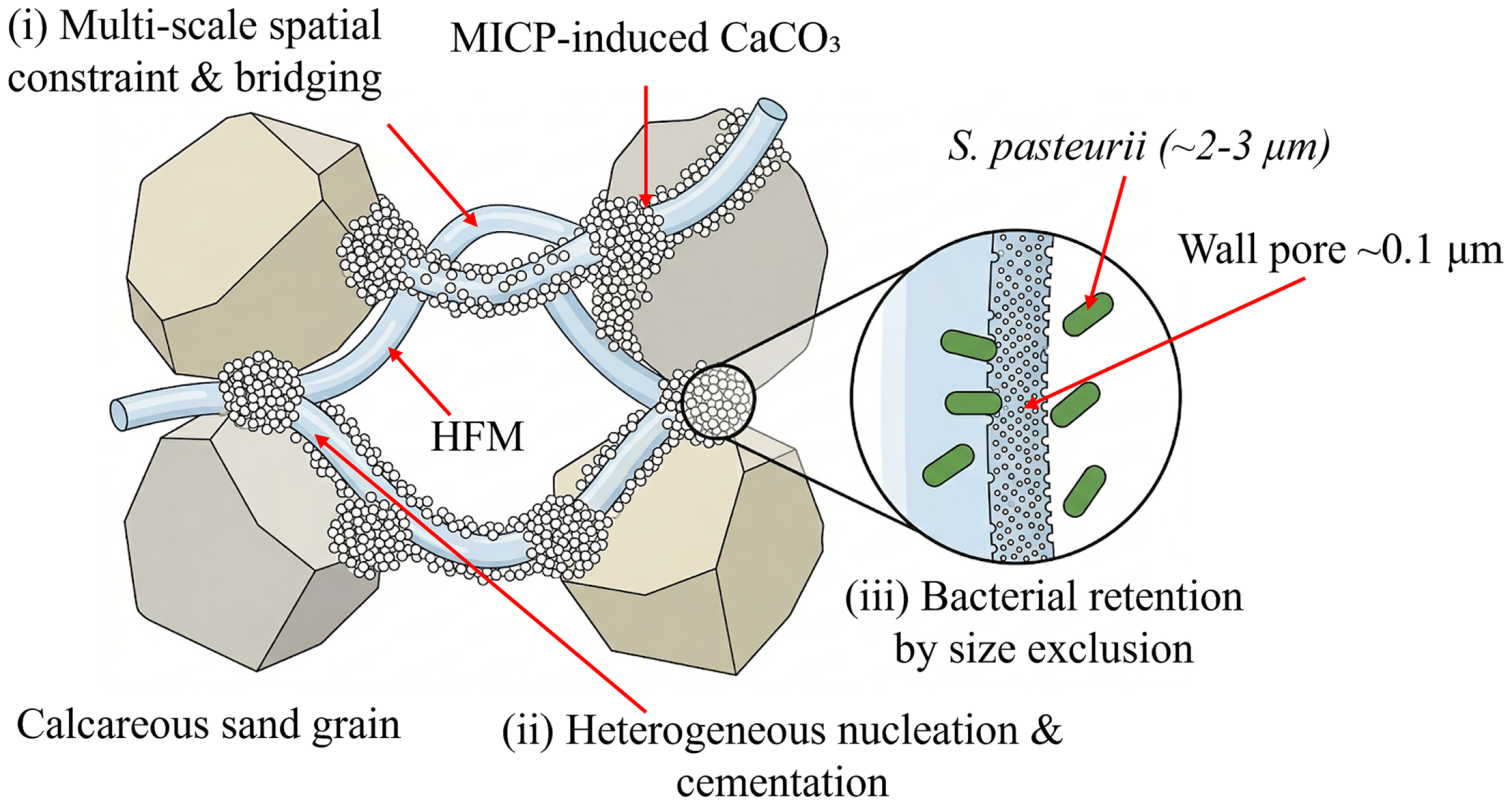
Coupled mechanisms of HFM-assisted MICP reinforcement in calcareous sand.

The role of the HFM within these three actions can be summarised as follows (Figure 14). The HFM acts on bacterial movement by size exclusion, retaining *S. pasteurii* cells on its outer surface and within the inter-fibre void space, which localises high-density bacterial populations along the membrane–sand interface and elevates urease activity in this region. The locally elevated urease activity then drives CaCO₃ precipitation in the immediate vicinity of the membrane, so that the bridging crystals between non-contacting sand grains, the cementing deposits at sand–sand contacts, and the surface coatings on sand particles all preferentially nucleate where bacteria have been concentrated by the HFM. The HFM itself, threaded through the sand skeleton, additionally provides a multi-scale geometric constraint—at the fibre length scale (5–20 mm) it bridges across multiple sand grains, and at the wall-pore scale (~0.1 μm) it offers an irregular substrate for heterogeneous nucleation. The bridging, cementation and coating actions documented in Figure 13 are therefore not independent contributions but a coupled outcome of HFM-mediated bacterial retention, HFM-induced local supersaturation, and HFM-templated nucleation (Figure 14). Of these three roles, the bacterial-retention contribution is specific to the hollow-fibre geometry: a solid fibre of equivalent diameter could in principle contribute the bridging and surface-nucleation actions, but lacks the wall-pore selectivity that simultaneously confines bacterial cells while allowing free passage of urea, NH₄^+^, Ca^2+^, and CO₃^2−^; in this sense the size-exclusion retention effect identified here is a distinguishing feature of the hollow-fibre membrane rather than of fibrous reinforcement in general.

Definitively distinguishing CaCO₃ polymorphs (calcite, vaterite, and aragonite) requires crystallographic or spectroscopic evidence such as XRD, FTIR, or Raman spectroscopy, none of which were performed in the present study. We acknowledge this as a limitation of the work. One practical reason such phase analyses were not pursued is that the original calcareous sand already contains 62.9% CaCO₃ (Table 1), so newly precipitated MICP-derived CaCO₃ cannot be cleanly distinguished from the matrix background by bulk diffraction methods without dedicated sample-preparation protocols.

The crystals observed on the HFM surfaces in Figure 12 mainly show spherical and irregular habits rather than the well-formed rhombohedra typical of mature calcite. In MICP literature, spherical aggregates are often associated with vaterite or with metastable / transitional phases that may, given sufficient time and chemical conditions, partially convert to calcite. The present SEM evidence is therefore consistent with a still-evolving carbonate phase rather than a fully crystallised calcite product, and we deliberately refrain from claiming polymorph selectivity at this stage. Whether the confined microporous environment of the HFM ultimately drives the system toward calcite, and over what timescale, is an open question that XRD/FTIR/Raman analyses on matrix-corrected samples would be required to answer; we identify this as a priority direction for follow-up work.

While the morphological and crystallographic side of the precipitates is addressed above, the microbiological side of the proposed retention–colonisation–mineralisation pathway was likewise not characterised *in situ* inside the sand specimens. Direct techniques such as CFU enumeration, qPCR, and CLSM imaging are difficult to apply to the present specimens, in which an opaque, densely cemented matrix containing 62.9% native CaCO₃ obstructs un-scattered fluorescence imaging, while the mechanical disaggregation needed for plate counting or DNA extraction would destroy the spatial colonisation pattern that is the very feature of interest. The macroscopic indicators reported in Section 3.1, the 2.29-fold increase in bacterial-retention rate (12.33% → 28.27%) and the 37.7% increase in CaCO₃ generation rate (15.05%–20.72%) at the optimal HFM configuration, provide an aggregate trace of the same pathway, and the SEM observations in Figure 12 spatially localise the resulting precipitates onto the HFM surfaces. Quantitative spatial mapping using transparent microfluidic chips or simplified 2D analogues at the membrane–sand interface is left as the natural extension of this work.

## 4 Conclusion

This study combined MICP technology with hollow fiber membranes for reinforcing calcareous sand from South China Sea islands and reefs. By incorporating hollow fiber membranes of different contents (0.2–1%) and lengths (5–20 mm) into calcareous sand specimens, comprehensive investigations were conducted through physical property tests (calcium carbonate generation rate, water absorption rate, bacterial retention rate), mechanical property tests (unconfined compressive strength, unconsolidated undrained shear), and microscopic analyses. The study systematically examined changes in compressive and shear strength of calcareous sand before and after reinforcement, establishing optimal hollow fiber membrane content and length for microbial reinforcement applications. The following conclusions were drawn:

(1) Hollow fiber membranes significantly enhance MICP reinforcement effectiveness in physical properties. Under optimal conditions (0.6% content, 10 mm length), calcium carbonate generation rate reaches 20.72% (37.67% increase), bacterial retention rate achieves 28.27% (2.29-fold enhancement), and water absorption decreases to 16.62% (28.88% reduction). The microporous structure of hollow fiber membranes effectively retains bacteria, providing favorable biological conditions for MICP reactions and promoting calcium carbonate precipitation and pore filling.
(2) Mechanical properties exhibit substantial improvements. At optimal configurations, unconfined compressive strength reaches 1965.30 kPa (48.13% increase), while cohesion achieves 47.846 kPa (64.49% enhancement) compared to 12.07% improvement in internal friction angle. Results demonstrate that hollow fiber membranes primarily enhance shear strength through cohesion increases, attributed to calcium carbonate cementation effects and spatial network constraints.
(3) Significant interactions exist between hollow fiber membrane content and length. Longer membranes (20 mm) perform optimally at low contents (0.2–0.4%), moderate lengths (10–15 mm) excel at intermediate contents (0.5–0.6%), while shorter membranes (5 mm) prove most effective at high contents (0.7–1%). These patterns reflect fiber distribution characteristics and agglomeration effects within confined spaces.
(4) Microscopic analysis reveals triple reinforcement mechanisms: MICP strengthening through bridging, cementation, and coating actions; spatial constraints from hollow fiber membrane networks; and enhanced interfacial bonding from calcium carbonate crystallization on membrane surfaces. The composite “calcareous sand–calcium carbonate–fiber membrane” cementation system represents the key to strength enhancement.

The HFM-assisted MICP scheme explored here improves both the strength and treatment uniformity of reinforced calcareous sand under laboratory conditions, and offers a candidate route for the reinforcement of calcareous soils in coastal and island settings. Translating these laboratory results to field practice, however, will require validation under realistic marine conditions—including seawater salinity, elevated Mg/Ca ratios, and wet–dry cycling—which the present work did not reproduce. Future research should focus on field-scale validation, long-term durability assessment, construction process optimization, undrained shear testing under back-pressure saturation with B-value verification to characterise the saturated behavior of the reinforced sand, mineralogical and elemental characterisation of the precipitated CaCO₃ phase (XRD, FTIR, and EDS mapping) to identify polymorph selectivity at the membrane–sand interface, and environmental adaptability to promote practical implementation of this technology.

## Data Availability

The original contributions presented in the study are included in the article/supplementary material, further inquiries can be directed to the corresponding author.
